# Histopathological Features in a Case of Peters' Anomaly with Acquired Corneal Staphyloma

**DOI:** 10.1155/2011/418048

**Published:** 2011-12-22

**Authors:** Kumi Shirai, Yuka Okada, Yasushi Nakamura, Shizuya Saika

**Affiliations:** ^1^Department of Ophthalmology, Wakayama Medical University, 811-1 Kimiidera, Wakayama 641-0012, Japan; ^2^Department of Clinical Laboratory Medicine, Wakayama Medical University, 811-1 Kimiidera, Wakayama 641-0012, Japan

## Abstract

We report a case of corneal staphyloma histologically diagnosed as caused by Peters' anomaly. A 62-year-old male had a protruding opaque vascularized cornea that began to bulge from six months ago in the right eye. Since his right eye was blind and he wanted us to remove the eyeball for cosmetic improvement, we enucleated the affected eye. The enucleated tissue was fixed in formalin and embedded in paraffin for histological examination. Hematoxylin and eosin staining showed that the cornea lacked the posterior part of the corneal stroma and Descemet's membrane in the central region and the entire corneal endothelium. The corneal epithelium was keratinized. Collagen type I was strongly positive in peripheral cornea and weakly in protruding stroma. The cells labeled by antibodies against **α**SMA were scattered in the entire corneal stroma. As judged by the histological findings, the eye with the central corneal staphyloma was diagnosed as Peters' anomaly.

## 1. Introduction

Peters reported patients with central corneal opacity and ring-shaped iridocorneal adhesion caused by the absence of the Descemet's membrane [1]. This was named Peters' anomaly. Currently, Peters' anomaly was recognized as one of the mesenchymal dysgeneses of the anterior segments resulting from the abnormal development of neural crest cells [2]. On the other hand, the corneal staphyloma is characterized by a bulging cornea that was protruding anteriorly. On pathologic examination there is ectasia and the posterior aspect of the staphyloma is lined by uveal tissue [3]. Inflammatory cells are also often present within the cornea [4]. Peters' anomaly and corneal ulcer rarely complicate corneal staphyloma [5–9]. Although corneal staphyloma caused by Peters' anomaly is mainly detected in infant cases [5–8], here, we report an adult case of Peters' anomaly with a large corneal staphyloma diagnosed by histopathological examination.

## 2. Case Presentation

**Figure 1:**
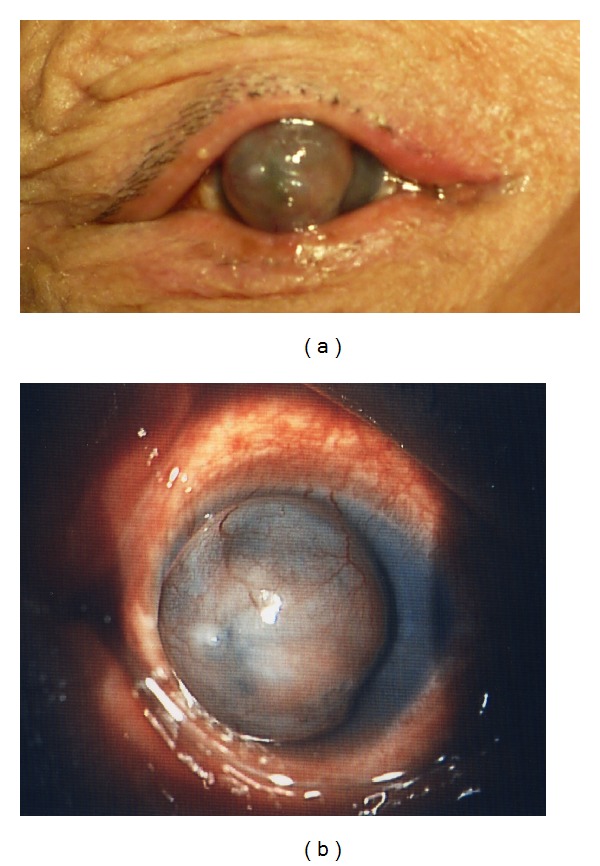
(a) The corneal bulge between the eyelids in a 62-year-old male. (b) Slit lamp photograph showed that the right cornea had the appearance of a smooth-surfaced opacified and vascularized protrusion bulging anteriorly.

A 62-year-old male was referred to our hospital with a protruding opaque cornea in the right eye. The patient told us that the central part of the cornea in his right eye had been opaque from his infancy but gradually began to bulge at 6 months before. At the first consultation we found that he could not blink because of markedly progressing corneal bulge (Figure 1(a)). His right eye was blind and the visual acuity of the left eye was 20/20.

**Figure 2:**
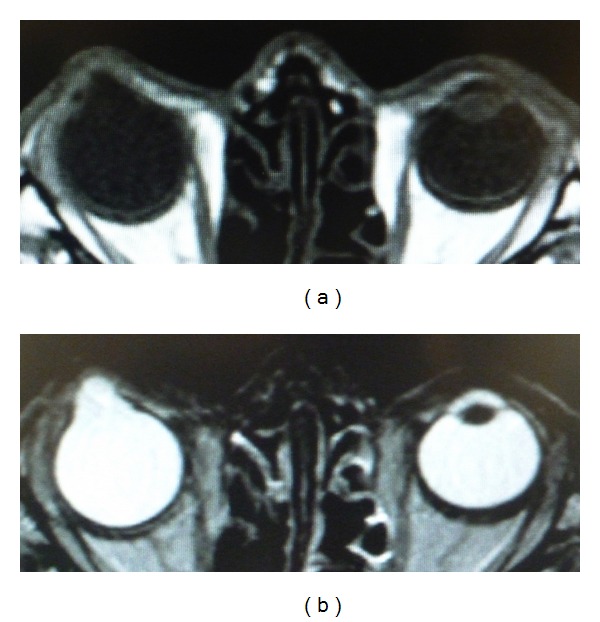
MRI image. The inside of the bulging lesion was not occupied with a tissue mass and lens was undetected. (a) T1, (b) T2.

Slit lamp examination showed that the right cornea had an appearance of a smooth-surfaced, opacified, and vascularized protrusion bulging anteriorly between the eyelids (Figure 1(b)). The corneal protrusion was 10 mm in its longitudinal diameter, 9 mm in its horizontal diameter, and 5 mm in its height. The iris was found to be fused with the posterior surface of the peripheral cornea with poor formation of the anterior chamber. Through the slit lamp the lens and ocular fundus in his right eye could not be observed. Intraocular pressure (IOP) was not be able to be measured accuracy in the right eye due to the severely protruded cornea. Magnetic resonance imaging (MRI) demonstrated that the inside of the bulging lesion was not occupied with a tissue mass and lens was undetected (Figure 2). Family history was unremarkable. No pathological findings were seen in the left eye.

**Figure 3:**
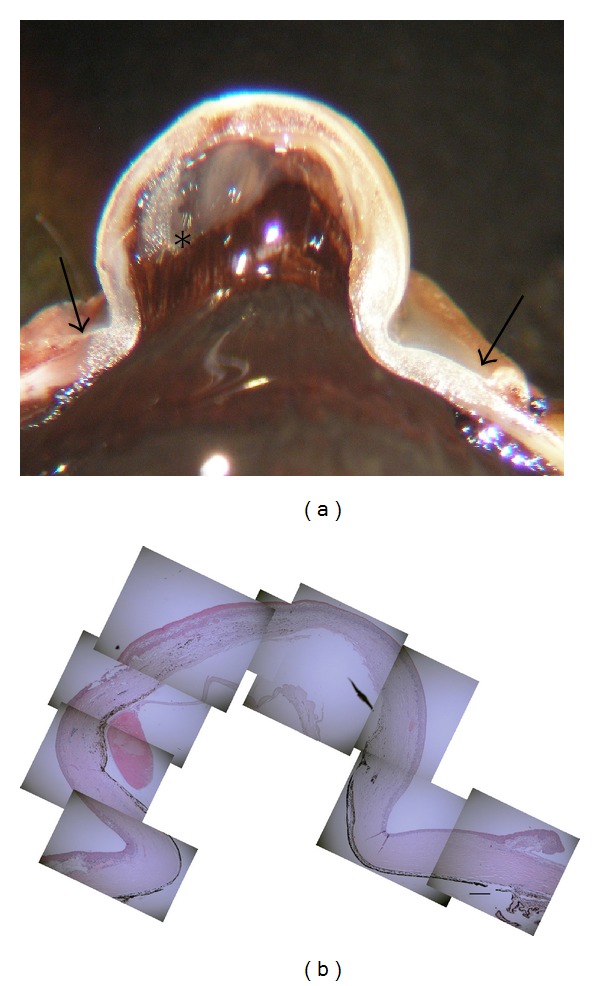
(a) Gross examination of the globe showed a large corneal staphyloma and very small remnant lens. Arrow: corneal limb. Asterisk; lens. (b) Histology of the corneal protrusion with HE staining. Bar: 300 *μ*m.

Clinically, the corneal protrusion in the right eye was thought to be a corneal staphyloma. Since his right eye was blind and thus he wanted us to remove the eyeball for cosmetic improvement, we enucleated the affected eye. Gross examination of the globe showed a large corneal staphyloma and very small remnant lens (Figure 3(a)). The retina was unremarkable.

We histologically examined the eyeball to investigate the cause of corneal staphyloma. Informed consent for the histological study of the enucleated eyeballs was obtained from the patient. The enucleated eye was fixed in 2.0% formalin and embedded in paraffin.

Immunohistochemistry was carried out to investigate the characteristics of the matrix of the staphylomatous tissue. Collagen I or IV is the major collagen type of the corneal stroma or epithelial basement membrane. *α*-smoooth muscle actin (*α*SMA) is the marker of the activation of the fibroblast or keratocyte. Deparaffinized sections were processed for hematoxylin and eosin (HE) staining and indirect immunohistochemical staining for goat polyclonal anti-type I collagen antibody (1 : 100; Southern Biotechnology Associates, Inc. Birmingham, AL, USA), goat polyclonal anti-type IV collagen antibody (1 : 100; Southern Biotechnology Associates, Inc. Birmingham, AL, USA), and mouse monoclonal anti-*α*SMA antibody (1 : 100; NeoMarker, Fremont, CA, USA). The specimens were then treated with appropriate peroxidase-conjugated secondary antibodies. The complex of the antibodies was visualized with diaminobenzidine reaction. The tissue was then counterstained with methylgreen.

**Figure 4:**
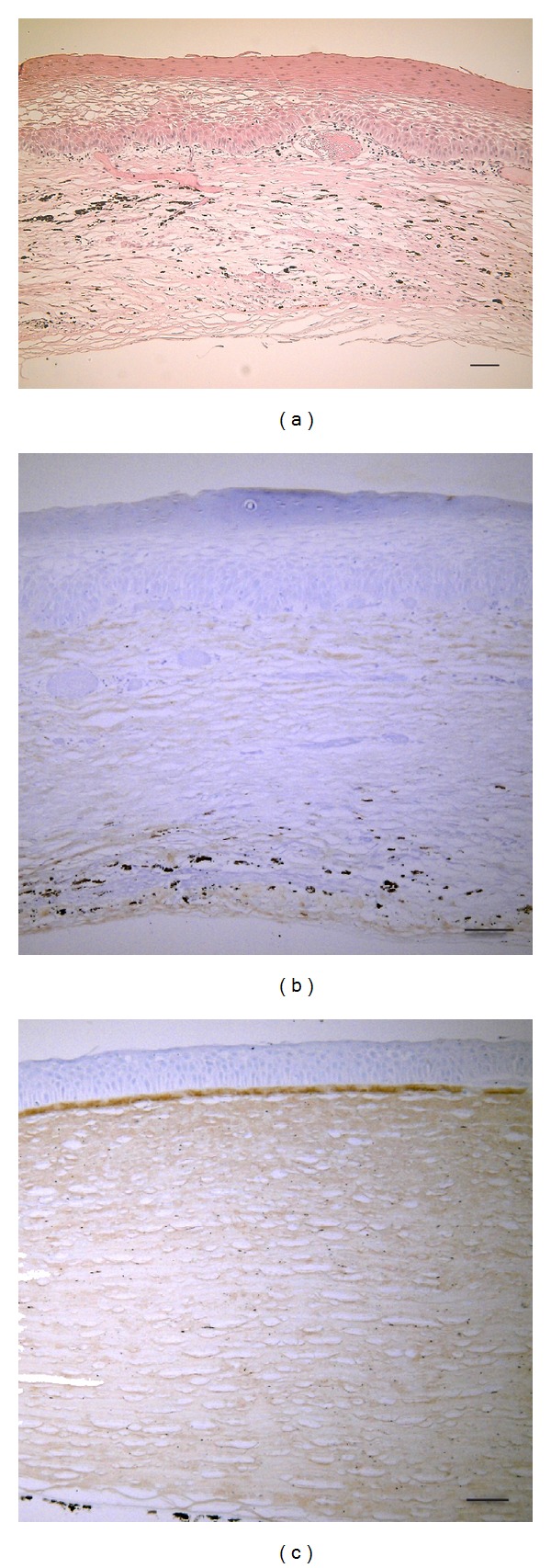
The central cornea with HE staining (a). The corneal epithelium was thickened and keratinized. Bowman's membrane was absent. The stroma was consisted of irregular collagen, fibroblast-like cells, pigmented cells, blood vessels, and presumed inflammatory cells. The corneal endothelium and Descemet's membrane were absent. Immunohistochemistry for Collagen types I (b) and (c). The protruding cornea (b). The peripheral cornea (c). Collagen type I was positive strongly in the peripheral cornea (c) and weakly in protruding cornea (b). Bar: 50 *μ*m.

**Figure 5:**
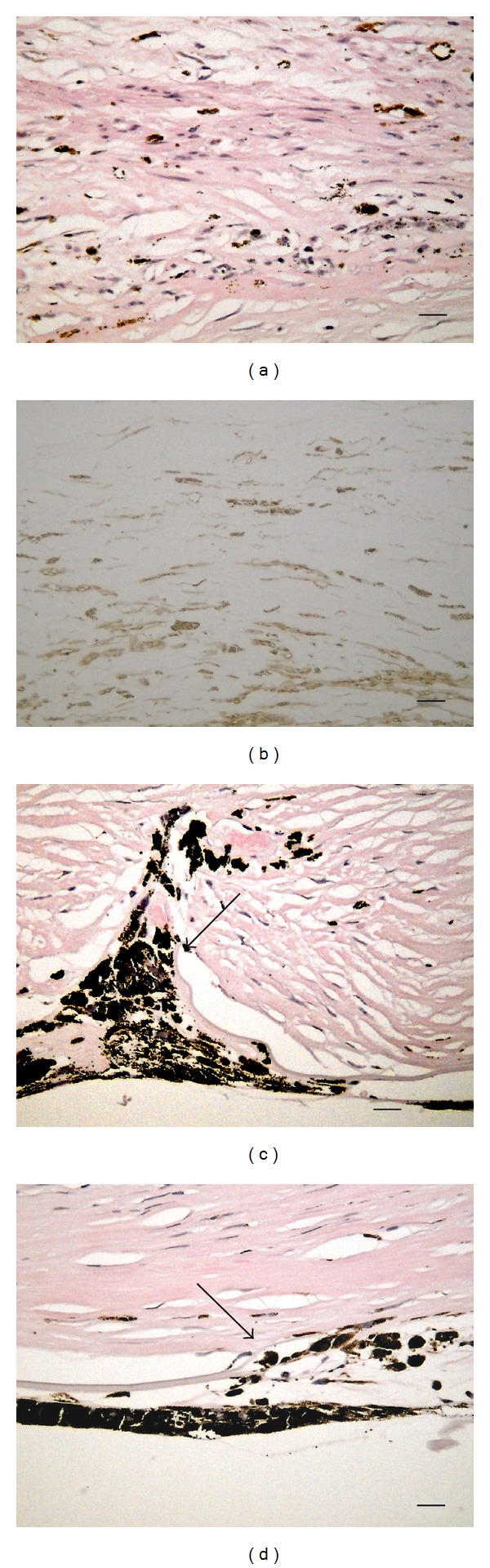
The corneal stroma with HE staining (a). The stroma was consisted of irregular collagen, fibroblast-like cells, pigmented cells, and presumed inflammatory cells. Immunohistochemistry for *α*SMA (b). The fibroblast-like cells labeled by antibodies against *α*SMA were scattered in corneal stroma. Posterior surface of the cornea with HE staining (c) and (d). Arrow: the end of Descemet's membrane. Bar: 10 *μ*m.

**Figure 6:**
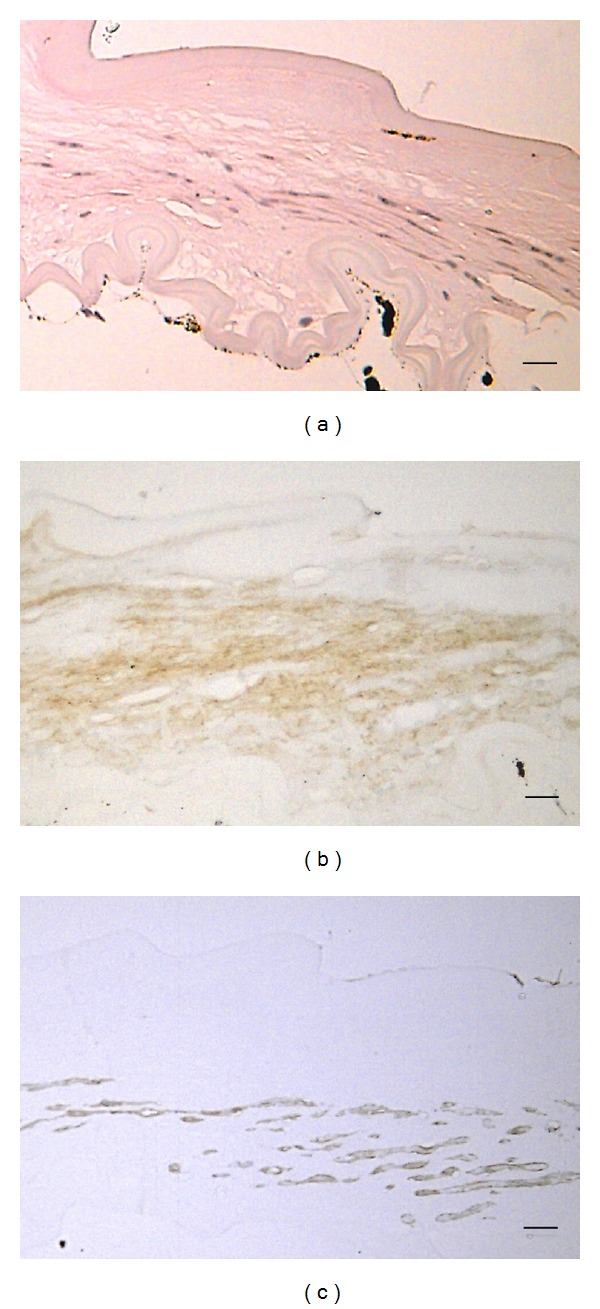
The remnant lens with HE staining (a). The contents of the crystalline lens were absorbed and fibrous tissue was present between anterior and posterior capsule. Immunohistochemistry for Collagen types I (b) and *α*SMA (c). Bar: 10 *μ*m.

 The corneal protrusion in the right eye was thought to be a corneal staphyloma (Figure 3(b)). Histology with HE staining revealed the following findings. The corneal epithelium was thickened and keratinized. Bowman's membrane was absent (Figure 4(a)). The stroma was consisted of irregular collagen, fibroblast-like cells, pigmented cells, blood vessels, and presumed inflammatory cells (Figures 4(a) and 5(a)). Cornea lacked entire corneal endothelium (Figure 4(a)). Descemet's membrane was absent in the region of the protruding cornea (Figure 4(a)). The end of Descemet's membrane was recognized (Figures 5(c) and 5(d)). The anterior chamber space was not formed and the iris stroma adhered to the posterior surface of the peripheral cornea (Figures 5(c) and 5(d)). The contents of the crystalline lens, that is, nucleus and lenticular fibers, were absorbed and fibrous tissue was present between anterior and posterior capsule (Figure 6(a)).

Immunohistochemistry revealed the following findings. Collagen type I was positive strongly in the peripheral cornea (Figure 4(c)) and weakly in protruding cornea (Figure 4(b)). The fibroblast-like cells labeled by antibodies against *α*SMA were scattered in corneal stroma (Figure 5(b)). The finding suggests the presence of the process of tissue repair. The fibrous tissue in lens was labeled by antibodies against collagen type I and *α*SMA (Figures 6(b) and 6(c)).

## 3. Discussion

In the present report we showed a case of an adult corneal staphyloma. Since his right eye was blind and thus he wanted us to remove the eyeball for cosmetic improvement, we enucleated the affected eye. We then investigated the histology of the cornea to clarify the cause of corneal staphyloma and concluded the case as caused by Peters' anomaly.

 Descemet's membrane and endothelium in the protruding cornea were absent. Crystalline lens was also found to be abnormally small. These findings were consistent with Peters' anomaly. The staphylomatous cornea was severely vascularized and keratinized in the superficial layer of the epithelium. Histology suggested the presence of secondary tissue repair-associated changes caused by an impaired eyelid closure. Myofibrobalsts were detected in the corneal stroma that suggested that the presence of the wound healing reaction has occurred previously. Type I collagen immunoreactivity was much less in the central region of the staphyloma, while the peripheral cornea contained abundant type I collagen. These findings also suggest the wound healing reaction, because the percentage of collagen type I is reportedly less in a primary healed corneal stroma as compared with the mature stromal matrix. Considering these findings, it was thought that ulceration of the central corneal stroma and inflammation developed prior to the formation of staphyloma.

 In the present case the abnormality in the crystalline lens indicated that the case was type II Peters' anomaly [10]. Type II Peters' anomaly exhibits abnormal crystalline lens that adhere to the posterior surface via lens capsule, while the type I case has an intact lens. In our case, the fibrous tissue in lens was labeled by antibodies against collagen type I. The fibroblast-like cells in the tissue were labeled by antibodies against *α*SMA. These findings confirmed epithelial mesenchymal transition of the lens epithelial cells, a process of transdifferentiation of lens cells.

 There is a possibility that the absence of endothelium and Descemet's membrane gradually led to staphyloma formation as a result of no resistance to raised intraocular pressure. Glaucoma is frequently present in Peters' anomaly, although in our case, IOP in right eye was impossible to measure. Matsubara et al. suggested that patients with Peters' anomaly should check the development of severe anterior staphyloma even when they have a normal IOP [8]. Corneal staphyloma is thought to be a terminal stage of mesenchymal dysgenesis of anterior segment. Many of reports of corneal staphyloma are regarding with congenital or infantile corneal staphyloma [5–8]. In the present case, histology and immunohistochemistry provided information toward the diagnosis of Peters' anomaly in a case of prominent corneal staphyloma.
